# When Natural Hazards Intersect with Public Health: A Preliminary Exploration of the Impact of Bushfires and the COVID-19 Pandemic on Australian Coastal Drowning Fatalities

**DOI:** 10.3390/ijerph18105314

**Published:** 2021-05-17

**Authors:** Jasmin C Lawes, Luke Strasiotto, Shane Daw, Amy E Peden

**Affiliations:** 1 Surf Life Saving Australia, Bondi Beach, Sydney, NSW 2026, Australia; lstrasiotto@slsa.asn.au (L.S.); sdaw@slsa.asn.au (S.D.); 2 School of Biological Earth and Environmental Sciences, UNSW Sydney, Sydney, NSW 2052, Australia; 3 Beach Safety Research Group, UNSW Sydney, Sydney, NSW 2052, Australia; a.peden@unsw.edu.au; 4 School of Population Health, Faculty of Medicine, UNSW Sydney, Sydney, NSW 2052, Australia

**Keywords:** natural hazards, drowning, bushfires, COVID-19, SARS-CoV-2, climate, pandemic, injury, risk reduction, safety

## Abstract

Natural hazards combined with the COVID-19 pandemic have had significant global impacts to the community and the environment. This study explores the impact of the Australian 2019/20 bushfires followed by the COVID-19 pandemic on unintentional coastal drowning fatalities. Fatality data were collated using triangulation methodology. Percentage change in coastal drowning fatalities between 2019/20 financial year (FY) and the 15FY annual average (2004/5–2018/19) were calculated for the dominant bushfire period (August 2019–February 2020 inclusive) and COVID-19 restrictions in place for 2019/20FY (March–June 2020 inclusive). Relative risk (RR; with 95% confidence intervals [CI]) of coastal drowning was calculated against the average for overall, bushfire and COVID-19 periods, using coastal participation data as the denominator, weighted for the predicted decrease in the use of outdoor coastal areas due to these widespread events. Coastal drowning fatalities increased in 2019/20FY by 9% overall (bushfires: 6%; COVID-19: 9%). Swimming/wading drowning fatalities increased during the bushfire period (RR = 2.02; 95% CI: 1.13–3.63), while boating and personal watercraft (PWC)-related fatalities increased during both the bushfire (RR = 2.92; 95% CI: 1.41–6.05) and COVID-19 period (RR = 3.86; 95% CI: 1.64–9.11). Rock fishing fatalities also increased across both the bushfire (RR = 4.19; 95% CI: 1.45–12.07; *p* = 0.008) and COVID-19 (RR = 3.8;95% CI: 1.24–11.62; *p* = 0.027) periods. Findings indicate the activity patterns leading to coastal drowning fatalities changed despite significant public health events impacting freedom of movement and thus opportunity for coastal participation. Understanding, and preparing for, the impacts of natural hazards on drowning risk is vital for future preventive efforts.

## 1. Introduction

Understanding impacts of natural hazards on the community and the environment is crucial for survival and has become increasingly challenging since the spread of the novel coronavirus disease (COVID-19) pandemic. COVID-19 has been described as the worst global crisis since World War II with socio-economic and geo-political consequences expected to persist for decades [1]. Pandemics are classified as natural hazards [1], where the hazard risks are shaped by the disaster type and vulnerability and exposure levels. However, how does this relationship change when multiple natural hazards occur simultaneously or subsequently, without time to recover? Understanding additive, synergistic, and interactive effects of natural hazards is crucial to predict changes to community behaviours and the environment so that they can be appropriately managed. Intersecting public health and environmental hazards should be considered collectively as implications are clearly connected [2]. For example, plastic pollution has increased significantly, with COVID-19 precautionary measures challenging environmental sustainability (especially within marine systems), highlighting the need to shift towards more sustainable alternatives and to promote both green and blue economies [2].

Drowning is a leading public health concern globally with an estimated 320,000 drowning deaths recorded each year [3]. In Australia, a significant contributor to the total drowning burden is coastal drowning with an average of 112 unintentional drowning deaths occurring in Australian coastal waters [4]; summer being a peak period of risk [5]. Traditionally in Australia, swimming and wading is the leading category of activity prior to unintentional coastal drowning [4], but the profile of coastal drowning during the most recent year (2019–2020 financial year [FY]) differed substantially, with boating and personal watercraft (PWC) recording the highest number of coastal drowning deaths [4] for the first time since formal records began in 2004. Anecdotally, this differing drowning profile has been attributed to the impact of two co-occurring public health and environmental concerns facing Australia during 2019–20 (bushfires and the COVID-19 pandemic) which are thought to have substantially altered people’s behaviour [6]. 

The additive effects of severe bushfires [7,8,9] and the similarly unprecedented COVID-19 pandemic have undoubtedly impacted Australia [10,11,12]. Together, these have resulted in exceptional restrictions and limitations to many Australian communities [13], including the closure of beaches [14] and changes to the use of existing coastal structures e.g., as locations of refuge for those displaced by bushfires [15]. The impact of COVID-19 was felt at a national level, while the majority of bushfire impacts were contained to the Eastern states of Australia (particularly New South Wales, Queensland, and Victoria). Satellite imagery [8] demonstrated that significant fires were spread across the nation with many residences and lives lost, such that the blanket of smoke covering the country was carried as far as New Zealand [12]. These two events have had widespread economic impacts with significant reductions in employment and business [16], especially in tourism and hospitality sectors, which are the dominant economic drivers for many coastal communities. These have been periods of intense change where many have worked from home and/or are now faced with financial hardship [11,16,17]. These stressors and hardship are likely to have encouraged behavioural changes in the use of coastal environments (e.g., increased fishing for food, or more time spent at the beach in response to changed employment circumstances) and which locations are frequented by beachgoers (i.e., beach closures resulting in visitors going to other aquatic locations). 

Surf Life Saving Australia (SLSA) is an iconic organisation and Australia’s peak coastal safety body, identified to provide community benefits worth $6.5 billion a year, with 90% of these benefits derived through Surf Life Saving’s coastal safety and lifesaving services that significantly reduce injuries and fatalities on our coast [18]. Surf Life Saving (SLS) services are tasked with protecting beachgoers, but, this year, SLS services were dramatically modified. During the bushfire period of impact, many surf lifesaving clubs were used as refuge areas for bushfire victims or were destroyed during the bushfires, and, in some cases, beaches were closed for lifesaving services due to the poor visibility and smoke conditions. Similarly, when COVID-19 restrictions began, in an attempt to protect the community and SLS members, some beaches were closed and services were altered to limit beach numbers and reduce community transmission of the virus [14]. Moreover, in an attempt to discourage people going to the beach, many surf lifesaving services were limited to non-contact surveillance only (i.e., using unmanned aerial vehicles [UAVs], helicopters, from lifeguard towers [i.e., with no patrol flags in place]) and reduced or ceased patrols completely, effectively ending the season early. With these measures in place, participation in coastal activities was expected to decrease, especially with respect to public health messaging in regards to outdoor usage under bushfire and smoke conditions [19,20] and COVID-19-related social distancing restrictions and lockdowns; however, these impacts were expected to vary with respect to locations and activity types. 

Considering that COVID-19 and similar geopolitical challenges with extensive environmental impacts are likely to be an ongoing concern, and that bushfire periods are increasingly more intense and unpredictable [9,12,21], this study explores using unintentional coastal drowning deaths to highlight impacts of these environmental stressors. Participation in coastal activities was unknown and therefore estimated to decrease in response to these stressors and associated restrictions; however, the level of impact was expected to vary and reflect different restrictions in place at different areas and regarding certain activities. Our secondary aim was to provide guidance to drowning prevention practitioners for the forthcoming, and future, summer periods, with respect to such culminating public health issues.

## 2. Materials and Methods

### 2.1. Study Design

We used a retrospective cohort study to explore the changing profile of coastal drowning under the impacts of two consecutive natural hazards occurring in Australia, the 2019/20 bushfires and the onset of the COVID-19 pandemic. The initial hypothesis, which has now been reinforced with a variety of literature [22,23,24,25,26,27], was that decreased outdoor activity due to the fires, smoke and COVID-19 restrictions would reduce unintentional drowning deaths. This hypothesis was developed such that decreased outdoor usage would reduce coastal participation (such as beach visitation) and result in a decreased risk of fatal coastal drowning. While the intersecting pressures of bushfires and COVID-19 influencing outdoor usage decreased, these two factors were investigated in combination because they shared the same predicted outcome (decreased coastal usage), occurred sequentially and may have had an additive effect. 

### 2.2. Procedures

To estimate changes to outdoor usage due to bushfires and COVID-19, percentage decreases were determined using various published resources [22,23,24,25,26,27,28]. It was estimated that the bushfires decreased outdoor activities by up to 54% [22,23] and that COVID-19 decreased outdoor activity (measured by proxies such as decreased use of parks, increased online shopping, decreased tourism; Table 1) by 39% [24,25,26,27] and an overall decrease of 49% for the total time period.

### 2.3. Data Sources

The primary data source for fatality data used in this study was the National Coronial Information System (NCIS), an electronic database of deaths notified to Australian Coroners from July 2000 (2001 in Queensland) and deaths notified to New Zealand Coroners from July 2007. The NCIS is an electronic database for which the Department of Justice and Community Safety is the source organisation of the data. Data on unintentional fatal coastal drowning were collated from the NCIS (including coroner’s findings, police narrative of circumstances, autopsy and toxicology reports), media reports and Surf Life Saving Australia’s SurfGuard Incident Report Database as per previous studies [29,30]. All Australian unintentional coastal drowning deaths (up to 12 nautical miles offshore) that occurred between 1 July 2004 and 30 June 2020 were included in the analysis. Financial years (as opposed to calendar years) are used by Surf Life Saving Australia (SLSA) for tracking year to year changes so the summer period (from December to February) in which drowning events historically peak has its data and therefore trends encapsulated in the same year.

### 2.4. Exploratory and Statistical Analyses

Differences observed in the 2019/20FY were compared against the annual average of coastal drowning deaths by month for the previous 15 reporting periods (2004/05–2018/19 financial years). The whole year, the dominant bushfire period (August 2019 to February 2020 inclusive) and the commencement of COVID-19 restrictions in Australia (March-June inclusive) were compared. Bushfire and COVID-19 periods of interest were determined using official reports and decisions made by governments and emergency services (e.g., declarations of state of emergency, travel bans, etc.) in the context of impacts on the Australian community. Although the decreased use of outdoor spaces in these events were predicted to result in a decrease in drownings, an increase in drownings from the 15FY average was observed. Consequently, the percentage decreases in outdoor activity use (Section 2.2) were applied to the historical drowning data to create a weighting to be applied to coastal usage data. Relative risk is a statistical analysis used to calculate the increased probability of an outcome (drowning death) due to exposure to a factor (bushfires and/or COVID-19). Relative risk was calculated in R (version 3.6.1) using the package “epitools.” The relative risk ratio analyses performed included 95% confidence intervals, with an increase in relative risk indicating an increased risk of drowning in the 2019/20 bushfire and COVID-19 periods. 

To be more specific, in regards to the relative risk format, drowning decedents were the exposed group within this study, while the unaffected control groups used were coastal participants (2019/20FY) and coastal participants multiplied by the appropriate estimated weightings (2004/05–2018/19FY) as described in Section 2.2. Coastal participation data were gathered through Surf Life Saving’s annual National Coastal Safety Surveys [31]. Due to the challenges of statistical methods with small numbers and single replicates, percent change and cumulative counts were calculated and used to describe and visualise observed trends and differences throughout the year and the determined periods of interest. These exploratory analyses were conducted for common coastal activities, locations and for each state/territory. Due to ethical constraints associated with small numbers, where table cell counts <5, NP (Not Presented) have been used.

### 2.5. Research Ethics

This study was conducted with ethics approval from the Department of Justice and Community Safety Human Research Ethics Committee (JHREC; CF/07/13632; CF/10/25053; CF/16/17314).

## 3. Results

Across the 16-year study period, there were 1744 coastal drowning deaths. One hundred and twenty coastal drowning deaths were recorded in 2019/20FY, a 9.1% increase on the annual average ($\bar{x}$ = 110, 15FY). Small percent increases were observed over both the bushfire (5.8%) and COVID-19 (8.6%) periods (Table 2). These increases meant that the 2019/20FY recorded significantly more drowning deaths than was expected given the estimated decreased outdoor usage levels. Specifically, the risk of drowning doubled (RR = 2.08 times; 95% CI = 1.61–2.7; *p* < 0.001; Table 3) in 2019/20, with drowning risk increasing 2.32 times (RR = 2.32; 95% CI = 1.67–3.24; *p* < 0.001) during the bushfire period and 1.75 times during COVID-19 (RR = 1.75; 95% CI = 1.11–2.77; *p* = 0.02; Table 3).

### 3.1. Coastal Activity Differences and Trends

**Figure 1:**
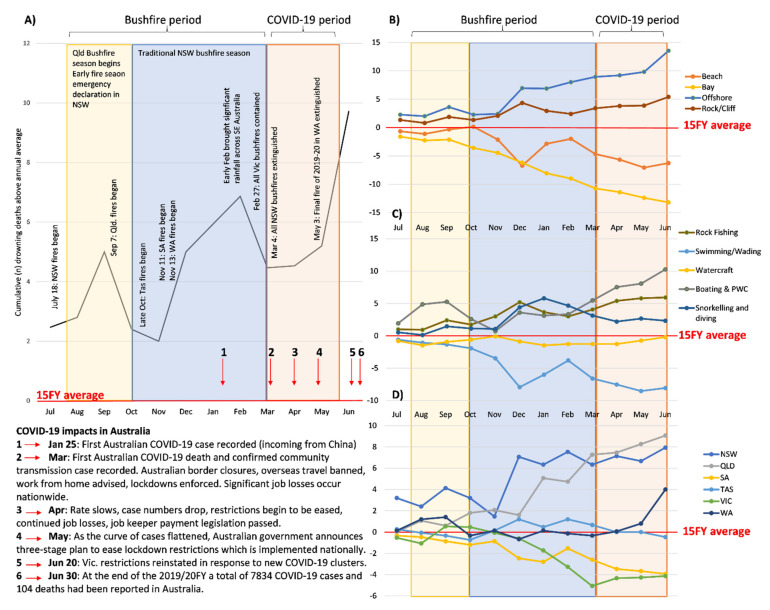
Cumulative differences between 2019/20 financial year compared to the 15-year (15FY) average by (**A**) coastal drowning with important dates relating to the bushfires and the arrival of COVID-19, (**B**) coastal location type, (**C**) activity, and (**D**) state. Shading represents significant time periods of interest.

Differences were observed for individual coastal activities (Figure 1). Cumulative trends show boating & PWC differed the most, with fatalities considerably above the 15FY average, followed by rock fishing (Figure 1). Snorkelling- and diving-related drowning deaths also increased, mainly during the bushfire period (Figure 1). Swimming/Wading drowning fatalities were considerably lower than the 15FY average, while watercraft drowning deaths remained in line with 15FY averages (Figure 1). Boating & PWC activities recorded the most drowning deaths, increasing by +41.7% in 2019/20 (*n* = 34) on the annual average ($\bar{x}$ = 24; Table 2). Increases were observed for both periods (bushfire period: +7.1%) but was substantially higher during the COVID-19 period (*n* = 15), +87.5% greater than the 15FY average for that period ($\bar{x}$ = 4; Table 2). Although swimming and wading activities recorded the second highest number of drowning deaths, this was a decrease of −24.2% in 2019/20 (*n* = 25) from the annual average ($\bar{x}$ = 33; Table 2). This decrease was also observed across both time periods but was more pronounced during the COVID-19 period where a −50.0% reduction (*n* = 4) from the 15FY average for that period ($\bar{x}$ = 8) was observed (Table 2). Rock fishing drowning deaths (*n* = 18) recorded a + 50.0% increase from the annual average ($\bar{x}$ = 12; Table 2); again, increases were observed across both time periods (bushfire period, *n* = 8, +33.3%), but a higher percentage change (+60%) was observed during the COVID-19 period (*n* = 8; $\bar{x}$ = 5; Table 2). Unpowered watercraft recorded eight drowning deaths, equal to the annual average ($\bar{x}$ = 8). No change was observed during the bushfire period (*n* = NP, 0.0%), but an increase of +33.3% was recorded during the COVID-19 period (*n* = NP; $\bar{x}$ = 3; Table 2). Snorkelling and diving activities recorded a 16.7% increase (*n* = 14; $\bar{x}$ = 12), but these were attributed to a +50% increase in incidents recorded during the bushfire period (*n* = 12; $\bar{x}$ = 8) while a considerable reduction (−66.7%) was observed during the COVID-19 period (*n* = NP; $\bar{x}$ = 3; Table 2).

Relative risk ratios were performed for the three activities responsible for the highest number of drowning deaths, comparing the historical average of drowning deaths had coastal participation decreased as expected (Table 3). The risk of drowning while swimming and wading did not increase for the entire year (overall) or during COVID-19 (Table 3). However, there was a 2.02 times greater risk of drowning from swimming during the bushfire season (RR = 2.02; 95% CI = 1.13–3.63; *p* = 0.02). There were increases in annual drowning risk related to rock fishing and boating & PWC activities, as well as both the bushfire and COVID-19 seasons (Table 3). The risk of drowning while rock fishing increased by 4.22 times (RR = 4.22; 95% CI = 2.03–8.75; *p* < 0.001) over the year, was 4.19 times greater during the bushfire season (RR = 4.19; 95% CI = 1.45–12.07; *p* = 0.008) and 3.8 times greater in the COVID-19 period (RR = 3.8; 95% CI = 1.24–11.62; *p* = 0.027). For boating, drowning risk increased by greater than three times over the whole year (RR = 3.45; 95% CI: 2.048–5.82; *p* < 0.001), more than double the risk during the bushfire season (RR = 2.95; 95% CI = 1.41–6.05; *p* = 0.005) and a 3.86 times increased risk during COVID-19 (RR = 3.86; 95% CI: 1.64–9.11; *p* < 0.002).

### 3.2. Location Differences and Trends

Cumulative trends show beaches and bays below the 15FY average, while rock/cliff locations and offshore waters were considerably above (Figure 1). Beaches remained the coastal location where most drowning deaths occurred (*n* = 45) yet recorded a −11.8% decrease from the annual average ($\bar{x}$ = 51). The bushfire period drowning deaths remained similar (*n* = 34, −2.9%; $\bar{x}$ = 35), but considerable decrease observed during the COVID-19 period (*n* = 10, −28.6%; $\bar{x}$ = 14). Bay locations demonstrated the same pattern as beaches (2019/20FY: *n* = 6, −25%; $\bar{x}$ = 8; Bushfire: *n* = 5, 0%; $\bar{x}$ = 5; COVID-19: *n* = NP, −66.7%; $\bar{x}$ = 3; Table 2). Offshore waters recorded the second highest number of coastal drowning deaths (*n* = 36), a + 56.5% increase from the annual average ($\bar{x}$ = 23; Table 2). Increases were observed for both periods but were higher during the COVID-19 period (*n* = 13), +85.7% above the 15FY average for that period ($\bar{x}$ = 7; Table 2). Rock/Cliff locations increased by 23.8% (*n* = 26; $\bar{x}$ = 26) in 2019/20. The bushfire period remained similar (*n* = 12, +9.1%; $\bar{x}$ = 11), but an increase was observed during COVID-19 period (*n* = 11, +37.5%; $\bar{x}$ = 8; Table 2). 

### 3.3. Jurisdictional Differences and Trends

The cumulative differences in coastal drowning deaths were also observed by state (Figure 1). Queensland recorded the largest increase in drowning deaths from the 15FY average (*n* = 29, +38.1%; $\bar{x}$ = 21), followed by New South Wales (*n* = 49, + 16.7%; $\bar{x}$ = 42) and Western Australia (*n* = 21, + 24%; $\bar{x}$ = 17). The largest decreases for the year were in Victoria (*n* = 12, −25%; $\bar{x}$ = 16), and South Australia (*n* = NP, −50%; $\bar{x}$ = 8; Table 2). Tasmania recorded no difference from the 15FY average (*n* = 5, 0%; $\bar{x}$ = 5; Table 2). During the bushfire period, drowning deaths differed by state, with notable increases recorded in Queensland (*n* = 17, 41.7%; $\bar{x}$ = 12), Tasmania (*n* = NP, +33%; $\bar{x}$ = 3) and New South Wales (*n* = 30, +15.4%; $\bar{x}$ = 26; Table 2). This contrasted to the decrease in drowning deaths seen in the 2019/20 bushfires for Victoria (*n* = 8, −27.3%; $\bar{x}$ = 11) and South Australia (*n* = NP, −20%; $\bar{x}$ = 5, Table 2). Western Australia had no change in drowning deaths during the bushfire period (*n* = 10, 0%, $\bar{x}$ = 10; Table 2). State trends also differed during the COVID-19 period up until June, with notable increases observed in Western Australia (*n* = 10, +66.7%; $\bar{x}$ = 6) and Queensland (*n* = 11, +57.1%; $\bar{x}$ = 7), while no change was observed in New South Wales (*n* = 13, 0%; $\bar{x}$ = 13; Table 2). There was a −20% decrease in drowning deaths recorded in Victoria (*n* = NP, $\bar{x}$ = 5), and a 100% decrease observed in South Australia (*n* = 0, −100%; $\bar{x}$ = 2) and Tasmania (*n* = 0, −100%; $\bar{x}$ = 2; Table 2). Northern Territory was excluded from analyses as there were no coastal drowning deaths recorded in 2019/20FY.

## 4. Discussion

Coastal drowning significantly contributes to the overall Australian drowning toll, with summer a peak period [4,5]. This study aimed to explore the profile of drowning at coastal locations in the 2019/20 FY to explore the impact of bushfires (and the associated smoke, beach closures and evacuations) and the COVID-19 pandemic. Analyses showed a shift in drowning deaths associated with certain activities, where increases were observed in boating, PWC and rock fishing activities—especially over the COVID-19 period. The combined activity grouping of snorkelling and diving was the only activity to increase over the bushfire period and swimming/wading drowning deaths were lower over the periods of interest. Beaches and bays observed decreases, while rocky cliffs and offshore waters saw increases—especially over the COVID-19 period, these trends are most likely driven by activity shifts observed.

Surf lifesaving (SLS) services were originally put in place to prevent drowning related to swimming and wading recreation (i.e., swim between the flags), but SLS services often extend well beyond this [4]. These exploratory analyses suggest that the changed participation, drowning surveillance and COVID-19 restrictions that were placed on beachgoers may have successfully decreased swimming-related drowning deaths over these high-risk periods.

However, our results suggest that Australians modified their activities and use of coastal areas, which may have placed them at greater risk. For example, despite many jurisdictions having restrictions in place, boat sales were reported to have increased with many suppliers depleting their stock [32]. Local maritime agencies (i.e., state and territory government agencies responsible for vehicle, vessel registrations, permits and licenses) reported substantial increases in boat sales and boat registrations in the year [33,34]. This may lead to more people with lower levels of experience heading out on to the water to avoid the smoke, fires, to socially distance or undertake recreational activities less limited by COVID-19 restrictions and legislations.

Rock fishing also increased, which may indicate an interplay of socio-economic factors given the changed employment conditions or, more likely, the choice of a (predominantly) recreational activity that enables social distancing and can provide an opportunistic meal. Given the often solitary nature of rock fishing (or fishing in general) as an activity, and its capacity as a natural food source, there were no specific restrictions placed on its participation (unlike beachgoing). Consequently, this allowed for rock fishing to be justified as an essential activity and therefore considered a perfect recreational activity to undertake under COVID-19 restrictions and potentially an alternative local recreational activity during the bushfire season with many holiday destinations unsafe to visit.

There were notable variations in drowning numbers by jurisdiction; however, although drowning numbers were expected to decrease due to the bushfires and COVID-19, this was only seen in South Australia and Victoria. Victoria was one of the worst affected states by the bushfires [12] and COVID-19 [35], although it is important to note that these data do not encapsulate the majority of Victoria’s second wave. However, this trend alone doesn’t explain the decrease in drowning deaths over this period as New South Wales being the other state most impacted by bushfires [12] and COVID-19 [35] saw an increase in drowning deaths during the bushfire season and no change in COVID-19. This difference may have been attributable to the decreased air quality in Victoria due to the bushfires compared to New South Wales.

During this time, there was a rise in drowning deaths in states less affected by both the bushfires [12] and COVID-19 [35] such as Western Australia and Queensland. It is posited that these observed increases in those less affected states may be due to changes to local coastal usage. For instance, the high prevalence of bushfires in New South Wales and Victoria meant that domestic interstate holidays from Western Australia/Queensland were decreased and instead opting for visits to their local coastline or domestic travel intrastate, and more people from interstate visiting. This is reinforced by Tourism Research Australia in which, for the states that had survey data available for all months, Western Australia, for example, had by far the smallest decrease in domestic arrivals and intrastate travel from January–July 2020 [36]. Queensland and Western Australia make up the largest coastline in Australia and a large portion of it is in rural regions with less access to emergency services—domestic travel within these states to these rural areas could confer a higher risk of drowning and current data for January–June 2020 shows that there was a smaller decrease in regional travel than travel to major cities [36]. This is suggestive of changes in travel behaviour on a state-by-state basis, which could also be indicative of potential changes in local recreational coastal usage in lieu of traditional travel holidays. While outside the scope of this paper, regionality of drowning showed a 3% increase in rural drowning deaths Australia-wide (unpublished data), although we propose this to be highlighted for future research opportunities. Similarly, Australia-wide COVID-19 restrictions were enacted in March including stay-at-home directives asking Australians to not leave the house for non-essential activities [35]; however, Queensland and Western Australia were comparatively unaffected by COVID-19. Consequently, individuals may have taken this opportunity where they were directed to not be at work to undertake local activities such as boating and fishing which could be justified as essential activities, and this increased exposure combined with redistributed services emergency services could have conferred to increased risk of drowning.

With the arrival of ‘La Nina’, 2020/21 is a completely different year meteorologically [7]; however, the threat of bushfires and the COVID-19 pandemic remain. This study provides insights into the potentially changing the profile of drowning in response to these additional public health threats. The change in coastal participation and thus the profile of coastal drowning fatalities suggest that prevention strategies need to be adaptive and responsive to broader issues. Examples from this study include the need to encourage increased use of lifejackets and safe boating practices such as the carry and use of Emergency Positioning Indicating Radio Beacon [EPIRBs], vessel maintenance and licensing checks. With the increased surveillance around social distancing, there may be opportunity to include policing and enforcement of other legislation that aids in reducing drowning risk such as compulsory lifejacket usage on designed ‘black spot’ rock platforms in New South Wales [35], while boating [37] or enforcing beach closures. The implementation of public rescue equipment at popular locations for high-risk activities such as rock fishing may also be beneficial in preventing fatalities. Similarly, for these high-risk areas beyond the traditional Surf Life Saving patrol regions, technology could potentially be developed for remote and automatic monitoring of incidents of people entering the water.

### Strengths and Limitations

This is one of the first explorations into multiple intersecting public health issues facing Australia (i.e., bushfires and the COVID-19 pandemic) and the first to investigate their impact on unintentional fatal drowning in the coastal environment. However, due to the lack of accurate exposure data for this short time period, our hypothesis driven analyses were performed using carefully developed estimates of the decrease in drowning. Even so, it is important to note that the 2019/20FY data and 15-year averages contain a complete collection of coastal drowning incidents and are a total population of fatal coastal drowning incidents in Australia. Consequently, it is reasonable to report that observed changes (such as the increased number of drowning both compared to the average and compared to the estimated drownings) reflect true patterns. The 2019/20FY summer was a notably hot summer and dry summer, which may have driven more people to the coast—however, human behaviours were also altered by the onset of intersecting natural hazards. The increasing in fatal drowning reported here shows that, in spite of the excessive heat, despite the poor air quality and fire concerns, Australians were still interacting with coastal environments, although data regarding public perceptions, use and behaviour of coastal environments, are currently unavailable. Similarly, while numbers of coastal drowning fatalities do fluctuate, and have generally been increasing slightly over the past 16 years with a notable increase recorded since 2015/16FY [4], it is important to note that the increase observed in the 2019/20FY bushfire and COVID-19 periods was greater and attributed to different coastal activities. This study makes assumptions, based on a single year of interest (2019/20), that changes in activity prior to fatal drowning may be due to the influence of natural hazards (i.e., bushfires and the COVID-19 pandemic). However, as these are hazards that Australia will continue to face, their impact on communities including coastal drowning risk warrants discussion and further exploration, along with other natural hazards, e.g., floods and impacts of changing climates.

## 5. Conclusions

Natural hazards combined with the COVID-19 pandemic have had significant global impacts to the community and the environment. This study explores the impact of the Australian 2019/20 bushfires followed by the COVID-19 pandemic on unintentional coastal drowning fatalities. Coastal drowning is a significant contributor to the total Australian drowning toll. Understanding interactions between natural hazards is crucial to predict and manage community and environmental changes. The connected implications of intersecting public health and environmental hazards need to be considered in combination [2], especially given the social, economic and geo-political consequences being experienced worldwide [1]. Exploratory, hypothesis driven studies spanning multiple disciplines are necessary for the sustainability of humans and the environment in which we live.

Drowning and other public health concerns may experience changing profiles (e.g., [6]), highlighting the global relevance of this study. This exploration of the impact of bushfires and the COVID-19 pandemic on Australian coastal drowning fatalities and identified significant changes in activities being undertaken prior to drowning. While swimming and wading fatalities showed a decline this year, the number of swimming/wading-related coastal drowning deaths still requires attention—especially as restrictions are lifted and climate conditions varied. There is a need to continue to push and deliver safety education around swimming at patrolled beaches, identifying rip currents and other general safe practices. However, the increase in boating and the purchase of recreational boating craft indicates that it is reasonable to expect there will be more craft users on the water; and that more efforts around safe operating practices for boating and PWC activities are warranted. Such findings indicate the need for drowning prevention advocates to consider the impact of broader public health issues on drowning risk. This includes drowning prevention strategies which go beyond traditional swim between the flag messaging and patrolling of the beach, including increased efforts targeted at rock fishers and offshore boaters, such as the importance of wearing lifejackets, carrying safety equipment, and making sure you clearly communicate intended locations and return time with others.

## Figures and Tables

**Table 1:** Literature and data used to determine outdoor activity behaviour change.

Factor	Percentage	Justification for Inclusion	Reference
Bushfire period	0.64	Rescheduling of outdoor activities	[22]
	0.28	Non-asthmatics staying indoors due to smoke	[23]
COVID-19 period	0.70	Decrease in park usage in Wave 1	[24]
	0.43	Tourism and hospitality decrease	[25]
	0.22	Arts and recreation decrease	[25]
	0.33	Change to online shopping	[26]
	0.33	Decrease in short term visitors	[27]

**Table 2:** Numbers and percentage change of drowning deaths recorded in three determined periods of interest compared with 15-year (15FY) average for Australian coastal jurisdictions

Variable	Category	Timeframe	Whole Year		Fire Period		COVID-19 Period	
			*n*	% Change	*n*	% Change	*n*	% Change
Total	Coastal drowning deaths	2019/20	120	9.1	73	5.8	38	8.6
		15FY average (2004–2019)	110	–	69	–	35	–
Activity	Swimming/Wading	2019/20	25	−24.2	21	−12.5	NP	−50.0
		15FY average (2004–2019)	33	–	24	–	8	–
	Boating & PWC	2019/20	34	41.7	15	7.1	15	87.5
		15FY average (2004–2019)	24	–	14	–	8	–
	Rock Fishing	2019/20	18	50.0	8	33.3	8	60.0
		15FY average (2004–2019)	12	–	6		5	–
	Watercraft	2019/20	8	0.0	NP	0.0	NP	33.3
		15FY average (2004–2019)	8	–	NP	–	NP	–
	Snorkelling & Diving	2019/20	14	16.7	12	50.0	NP	−66.7
		15FY average (2004–2019)	12	–	8	–	NP	–
Location	Beach	2019/20	45	−11.8	34	−2.9	10	−28.6
		15FY average (2004–2019)	51	–	35	–	14	–
	Bay	2019/20	6	−25.0	5	0.0	NP	−66.7
		15FY average (2004–2019)	8	–	5	–	NP	–
	Offshore	2019/20	36	56.5	19	46.2	13	85.7
		15FY average (2004–2019)	23	–	13	–	7	–
	Rock/Cliff	2019/20	26	23.8	12	9.1	11	37.5
		15FY average (2004–2019)	21	–	11	–	8	–
Jurisdiction	New South Wales (NSW)	2019/20	49	16.7	30	15.4	13	0.0
		15FY average (2004–2019)	42	–	26	–	13	–
	Queensland (QLD)	2019/20	29	38.1	17	41.7	11	57.1
		15FY average (2004–2019)	21	–	12	–	7	–
	South Australia (SA)	2019/20	NP	−50.0	NP	−20.0	0	−100.0
		15FY average (2004–2019)	8	–	5	–	NP	–
	Tasmania (TAS)	2019/20	5	0.0	NP	33.3	0	−100.0
		15FY average (2004–2019)	5	–	NP	–	NP	–
	Victoria (VIC)	2019/20	12	−25.0	8	−27.3	NP	−20.0
		15FY average (2004–2019)	16	–	11	–	5	–
	Western Australia (WA)	2019/20	21	23.5	10	0.0	10	66.7
		15FY average (2004–2019)	17	–	10	–	6	–

Note: PWC = Personal Watercraft. NP = Not Presented.

**Table 3:** Relative risk analyses for test periods of overall coastal drowning and the top three coastal activities.

Variable	Time Period	Drowning Deaths (*n*)	Participation (*n*/1000)	Weighted Participation (*n*/1000)	RR	95% CI	*p*-Value
All activities	Annual average (12 months)	110	13,830	13,830	1	(1.61–2.7)	**<0.001**
	2019/20 (12 months)	120	14,097	7245	2.08		
	Average bushfire period	69	13,830	13,830	1	(1.67–3.24)	**<0.001**
	2019/20 bushfire period	73	14,097	6484	2.32		
	Average COVID-19 period	35	13,830	13,830	1	(1.11–2.77)	**0.02**
	2019/20 COVID-19 period	38	14,097	8570	1.75		
Activity							
Swimming/Wading	Annual average (12 months)	33	10,377	10,377.394	1	(0.93–2.63)	0.37
	2019/20 (12 months)	25	9763	5018.213	1.57		
	Average bushfire period	24	10,377	10,377.394	1	(1.13–3.63)	**0.02**
	2019/20 bushfire period	21	9763	4491.008	2.02		
	Average COVID-19 period	8	10,377	10,377.394	1	(0.26–2.9)	1.00
	2019/20 COVID-19 period	NP	9763	5935.941	0.87		
Boating & PWC	Annual average (12 months)	24	3182	3182.319	1	(2.05–5.82)	**<0.001**
	2019/20 (12 months)	34	2540	2539.674	3.45		
	Average bushfire period	14	3182	3182.319	1	(1.41–6.05)	**<0.005**
	2019/20 bushfire period	15	2540	2539.674	2.92		
	Average COVID-19 period	8	3182	3182.319	1	(1.64–9.11)	**<0.002**
	2019/20 COVID-19 period	15	2540	2539.674	3.86		
Rock Fishing	Annual average (12 months)	12	1224	1223.986	1	(2.03–8.75)	**<0.001**
	2019/20 (12 months)	18	854	854.144	4.22		
	Average bushfire period	6	1224	1223.986	1	(1.45–12.07)	**0.008**
	2019/20 bushfire period	8	854	854.144	4.19		
	Average COVID-19 period	5	1224	1223.986	1	(1.24–11.62)	**0.027**
	2019/20 COVID-19 period	8	854	854.144	3.80		

NB: Numbers of bushfire and COVID-19 drowning deaths will not equal total drowning deaths (*n*) since July 2019 is not included in either bushfire or COVID-19 periods. NP = Not Presented. Bold type indicates significance.

## Data Availability

Due to ethical requirements of this study, raw data can only be accessed by approved persons and cannot be publicly available. Approved persons may contact the authors to access the dataset.
